# Identification of four key prognostic genes and three potential drugs in human papillomavirus negative head and neck squamous cell carcinoma

**DOI:** 10.1186/s12935-021-01863-6

**Published:** 2021-03-12

**Authors:** Guocai Tian, You Fu, Dahe Zhang, Jiang Li, Zhiyuan Zhang, Xi Yang

**Affiliations:** 1 Department of Oral and Maxillofacial-Head Neck Oncology, Shanghai Ninth People’s Hospital, College of Stomatology, Shanghai Jiao Tong University School of Medicine, Shanghai, People’s Republic of China; 2 National Clinical Research Center for Oral Diseases, Shanghai, People’s Republic of China; 3 Shanghai Key Laboratory of Stomatology and Shanghai Research Institute of Stomatology, Shanghai, People’s Republic of China; 4 Research Unit of Oral and Maxillofacial Regenerative Medicine, Chinese Academy of Medical Sciences, Shanghai, People’s Republic of China; 5 Department of Oral Pathology, Ninth People’s Hospital, Shanghai Jiao Tong University School of Medicine, Shanghai, People’s Republic of China

**Keywords:** Head and neck squamous cell carcinoma, Human papillomavirus, Prognosis, Biomarker, Small molecule drugs, NVP-AUY922

## Abstract

**Background:**

Head and neck squamous cell carcinoma (HNSCC) is a common tumor worldwide with poor prognosis. The pathogenesis of human papillomavirus (HPV)-positive and HPV-negative HNSCCs differs. However, few studies have considered the HPV status when identifying biomarkers for HNSCC. Thus, the identification of biomarkers for HPV-positive and HPV-negative HNSCCs is urgently needed.

**Methods:**

Three microarray datasets from Gene Expression Omnibus (GEO) were analyzed, and the differentially expressed genes (DEGs) were obtained. Then, functional enrichment pathway analysis was performed and protein–protein interaction (PPI) networks were constructed. The expression of hub genes at both the mRNA and protein level was determined in Oncomine, The Cancer Genome Atlas (TCGA) and the Human Protein Atlas (HPA). In addition, survival analysis of the patient stratified by HPV status and the expression levels of key genes were performed based on TCGA data. The role of AREG, STAG3, CAV1 and C19orf57 in cancer were analyzed through Gene set enrichment analysis (GSEA). The top ten small molecule drugs were identified and the therapeutic value of zonisamide, NVP-AUY922, PP-2 and fostamatinib was further evaluated in six HPV-negative HNSCC cell lines. Finally, the therapeutic value of NVP-AUY922 was tested in vivo based on three HPV-negative HNSCC models, and statistical analysis was performed.

**Results:**

In total, 47 DEGs were obtained, 11 of which were identified as hub genes. Biological process analysis indicated that the hub genes were associated with the G1/S transition of the mitotic cell cycle. Survival analysis uncovered that the prognostic value of AREG, STAG3, C19orf57 and CAV1 differed between HPV-positive and HPV-negative patients. Gene set enrichment analysis (GSEA) showed the role of AREG, STAG3 and CAV1 in dysregulated pathways of tumor. Ten small molecules were identified as potential drugs specifically for HPV-positive or HPV-negative patients; three—NVP-AUY922, fostamatinib and PP-2—greatly inhibited the proliferation of six HPV-negative HNSCC cell lines in vitro, and NVP-AUY922 inhibited three HPV-negative HNSCC xenografts in vivo.

**Conclusions:**

In conclusion, AREG, STAG3, C19orf57 and CAV1 are key prognostic factors and potential therapeutic targets in HPV-negative HNSCC. NVP-AUY922, fostamatinib and PP-2 may be effective drugs for HPV-negative HNSCC.

**Supplementary Information:**

The online version contains supplementary material available at 10.1186/s12935-021-01863-6.

## Background

Head and neck cancer is the ninth most common among all malignancies worldwide [1]. Head and neck squamous cell carcinomas (HNSCCs) account for ~ 90% of all head and neck cancers [2]. Despite considerable efforts, the 5-year overall survival (OS) rate remains at 33–71%. Local recurrence, distant metastasis, and drug resistance are the main causes of death [3]. Approximately 75% of HNSCCs are associated with the consumption of tobacco and alcohol; however, some HNSCCs, particularly oropharyngeal tumors, are caused by human papillomavirus (HPV) infection [4].

HPV is a DNA virus that can infect skin and mucous membranes [5]. HNSCCs are divided into HPV-related HNSCCs and HPV-unrelated HNSCCs according to the infection status of HPV. HPV-unrelated HNSCCs have a worse therapeutic response than HPV-related HNSCCs [6]. To determine the pathogenesis of HNSCC, some biomarkers have been identified. However, few studies have considered the HPV status, which may cause unreliable results. Thus, the identification of reliable markers for HPV-positive and HPV-negative tumors is urgently needed to establish effective diagnostic, prognostic, and therapeutic strategies.

During the past years, integrated bioinformatic methods have been established to analyze high-throughput data across platforms, which helps us to identify differentially expressed genes (DEGs) and their related functional pathways involved in HNSCC carcinogenesis and progression. Gene expression omnibus (GEO) contains a large of high-throughput functional genomic datasets [7]. Some key genes in HNSCC, such as FN1, APP, SERPINE1, PLAU and ACTA1, were identified through GEO datasets [8, 9].

This study aimed to identify biomarkers for HPV-positive as well as HPV-negative HNSCCs via integrated bioinformatic methods. To avoid bias, DEGs were screened in three GEO datasets. Then Gene Ontology (GO) term and Kyoto Encyclopedia of Genes and Genomes (KEGG) pathway enrichment analyses were performed and protein–protein interaction (PPI) networks were constructed to analyze the difference in the molecular mechanisms active in HPV-positive and HPV-negative HNSCC tissues. The hub genes were determined, and survival analysis suggested that amphiregulin (AREG), stromal antigen 3 (STAG3), chromosome 19 open reading frame 57 (C19orf57) and caveolin-1 (CAV1) are key prognostic biomarkers for HPV-negative HNSCCs. Gene set enrichment analysis showed the role of AREG, STAG3 and CAV1 in dysregulated pathways of tumor. Finally, 10 small molecules were screened as potential drugs. The therapeutic value of zonisamide, NVP-AUY922, PP-2 and fostamatinib was further evaluated in six HPV-negative HNSCC cell lines, and NVP-AUY922 was tested therapeutic value in three HPV-negative HNSCC xenografts in vivo.

## Methods

### Acquisition of the datasets and identification of the DEGs

Datasets were searched in GEO (http://www.ncbi.nlm.nih.gov/geo) and the three datasets with the largest sample size (GSE39366, GSE40774, GSE55550 [10–12]) were sorted for further analysis. GEO2R is a web analysis tool for identifying DEGs across different experimental conditions. HPV 16-positive and HPV 16-negative samples (the HPV-inactive [DNA + RNA −] samples in GSE55550 were excluded to obtain the most significant results) were separated into two groups and analyzed with GEO2R. The Benjamini-Hochberg (false discovery rate, FDR) method was applied to adjust the P-values (adjusted P-value, adj.P-value). The probe identifiers were converted to gene symbols, and gene symbols with duplication or loss were deleted. The DEGs meeting the criteria of an adj.P-value of < 0.05 and a |log (FC)| of ≥ 1 were considered statistically significant DEGs. Metascape (https://metascape.org) is a web-based portal designed to provide a comprehensive gene list annotation and analysis resource [13]. Three groups of DEGs were uploaded to Metascape for meta-analysis to explore the most highly enriched pathways and the overlapping genes of the three datasets were visualized.

### Go term and KEGG pathway enrichment analyses

The GO database is the largest database worldwide that provides information on the functions of genes, including their biological processes (BPs), cellular components (CCs) and molecular functions (MFs) [14]. KEGG is a web database for exploring advanced functions of biological systems [15]. The Database for Annotation, Visualization and Integrated Discovery (DAVID, https://david.ncifcrf.gov) tool is an online tool comprising integrated biological knowledge bases and analytical tools [16]. The overlapping DEGs were submitted to GO and KEGG pathway analyses by DAVID (version 6.8). To identify the direct interactions among the DEGs, “GOTERM_DIRECT” BP, CC and MF categories were selected, and a P-value of < 0.05 was considered statistically significant.

### Construction of the PPI network and identification of hub genes

The Search Tool for the Retrieval of Interacting Genes/Proteins (STRING, https://string-db.org/) database is widely used to predict PPI networks, as a biological database, it integrates PPI information from publicly available sources [17]. To examine the functional connections among the DEGs, STRING (version 11.0) was used to determine the direct interactions among the DEGs. An interaction score of > 0.4 was considered statistically significant. The PPI network was visualized by platform Cytoscape (version 3.8.0) [18]. The Cytoscape plugin Molecular Complex Detection (MCODE) [19] was used to identify the top two modules in the PPI network with the following parameters: degree cutoff = 2, node density cutoff = 0.2, node score cutoff = 0.1, K-core = 2, and max.depth = 100. In addition, the cytoHubba plugin was used to calculate the degree of each protein node, and the top 11 genes were identified as hub genes. The expression of these hub genes in the three GSE datasets was visualized with R software (version 3.6.3).

### Verification of hub gene mRNA expression levels

Oncomine (https://www.oncomine.org/) is a cancer microarray database and integrated data mining platform [20]. The mRNA expression levels of the 11 hub genes were verified in Oncomine, and only two HNSCC datasets (Zhai Cervix [21] and Slebos Head-Neck [22]) met the criteria of including the HPV infection status. Differential gene expression was visualized with the R software.

The Cancer Genome Atlas (TCGA, https://www.cancer.gov/tcga.) contains many patient samples across 33 cancer types, with clinical information as well as genome sequencing data. In addition, University of California Santa Cruz (UCSC) Xena (https://xenabrowser.net/) is an online database that could be used to explore correlations between genomic and phenotypic variables [23], and TCGA data are included. To verify mRNA expression, HNSCC samples and mRNA expression data of the hub genes were downloaded from TCGA by UCSC Xena. Data for 604 samples, including HNSCC and solid normal tissues were obtained. Samples without mRNA expression data were removed, and the differential expression of the key genes was analyzed using GraphPad Prism software (version 8.0.0). The Mann–Whitney *U* test was performed, and a two-tailed exact P-value of < 0.05 was considered statistically significant.

### Immunohistochemical analysis

The Human Protein Atlas (HPA, http://www.proteinatlas.org, version 19.3) is the largest database mapping human proteins [24]. Since HPV status data of HNSCC samples were not available in the HPA, immunohistochemical data for the key genes in HPV-positive and HPV-negative samples were not provided. Thus, immunohistochemical analysis of HNSCC and normal tissues (all normal tissues were oral mucosal tissues) was performed.

### Survival analysis based on TCGA database data

Clinical information for the patients was downloaded from UCSC Xena, and the OS of patients stratified by the mRNA levels of the 11 key genes was analyzed. High expression or low expression was defined as an expression level of greater than or less than the median value, respectively. A log-rank (Mantel-Cox) test was performed, and a P-value of < 0.05 was considered to indicate a significant difference. For the comparison between the HPV-positive (+) and the HPV-negative (−) groups, the hazard ratio (HR) was calculated by the log-rank test as follows: HR = expression in the HPV (+) group/expression in the HPV (−) group. For comparisons between other groups, the HR was calculated as follows: HR = high expression/low expression. The disease-free interval (DFI), disease-specific survival (DSS) and progression-free interval (PFI) of patients stratified by the expression levels of genes with statistical significance in the OS analysis were analyzed.

### GSEA of AREG, CAV1, STAG3 and C19orf57

GSEA software (version 4.0.3, https://www.gsea-msigdb.org/gsea/downloads.jsp) was used to explore the association between gene expression and dysregulated pathways in cancer. The 73 samples from the TCGA database (HPV-negative) were divided into high and low expression groups based on the levels of AREG, CAV1, STAG3 and C19orf57, with the median value of each gene signifying the cutoff value. The predefined gene sets “c2.all.v7.1.symbols.gmt”, “c4.all.v7.1.symbols.gmt” and “c6.all.v7.1symbols.gmt” from the Molecular Signatures Database (MSigDB) were used for analyses. The normalized enrichment score (NES) was calculated, the cutoff for significance was defined as follows: a nominal p-value of < 0.05 and FDR q-value of < 0.25.

### Identification of potential small molecule drugs

The Connectivity Map (https://clue.io/cmap) is a resource that uses cellular responses to perturbations to find relationships between diseases, genes, and therapeutics. The CLUE platform (https://clue.io) was used to identify potential small molecules based on data from 9 tumor cell lines [25]. The DEGs co-expressed in the three GEO datasets were divided into up- and downregulated genes and were then uploaded to run the query. The parameters were defined as follows: gene expression (L1000) and Touchstone. After calculation, each small molecule was assigned an enrichment score ranging from − 100 to 100. A positive score indicates similarity between the signature of a given perturbagen and that of the query, while a negative score indicates a disparity between the two signatures. Scores of + 90 or higher and of − 90 or lower were considered strong scores for developing hypotheses for further study. Here, the molecules with the top five positive and negative scores were identified for further study. The 2D structures of these molecules are available in PubChem (https://pubchem.ncbi.nlm.nih.gov/), a public database of small molecules and their biological properties [26].

### Evaluation of the therapeutic value of small molecule drugs in vitro

HN4, HN6, HN30, SCC9, SCC25 and CAL27 cell lines were obtained from the Shanghai Key Laboratory of Stomatology and not contaminated with other cell lines by short tandem repeat analysis; zonisamide (Product ID: abs816590), NVP-AUY922 (Product ID: abs812121), PP-2 (Product ID: abs810398) and fostamatinib (Product ID: abs813307) were purchased from Absin Bioscience Inc. Zonisamide, PP-2, NVP-AUY922 and fostamatinib were dissolved in Dimethyl sulfoxide (DMSO) and diluted with medium to make final DMSO concentration < 0.002% with the medium. Approximately 3 × 10^3^ cells per well were seeded in 96-well culture plates. After culture for 24 h, the medium was removed. The four drugs at different concentrations were added with the volume of 100 µl. For the negative control wells, drug-free medium was added. In addition, the background control wells were added only drug-free medium but without cells. After culture for 72 h, the medium was removed, 100 µl of fresh medium containing 10% Cell Counting Kit-8 (CCK8, Dojindo, Japan) solution was added. The medium in the control wells was replaced by fresh medium. After incubation for 3 h, the absorbance at 450 nm was measured in a multimode microplate reader (SpectraMax i3, Molecular Devices). The experiments were repeated in triplicate. Finally, the cell inhibition rate was calculated with the following formula: %cell inhibition rate = 100 −  (mean optical density (OD) of the treatment wells −  mean OD of the background control wells)/(mean OD of the negative control wells – mean OD of the background control wells) × 100, and the half-maximal inhibitory concentrations (IC50) of the drugs were calculated with GraphPad Prism software (version 8.0.0, GraphPad Software, Inc. San Diego, CA, USA).

### In vivo studies

Female BALB/c nude mice (6 weeks of age, weighing ~ 20 g) were bred in SPF facilities of Shanghai Ninth People’s hospital. The experiment procedures were approved by the Laboratory Animal Care and Use Committees of the hospital. In the present study, HNSCC tumor models were established by inoculation 2 × 10^6^ HN6, SCC9, and SCC25 cells into the dorsal flank of the mice. When the tumor size reached about 5 mm, the mice were randomly assigned into control and NVP-AUY922 treatment group (n = 3 per group). Vehicle (10% DMSO, 5% Tween 20, 85% saline) and 50 mg/kg NVP-AUY922 were given intraperitoneal to each group three times a week for two weeks, and tumor size were measured by vernier calipers three times a week. The tumor volumes were calculated with the following formula: (length × width^2^)/2. Mice were sacrificed after 2 weeks.

### Statistical analysis

GraphPad Prism software (version 8.0.0) was used for statistical analysis and generating figures. To evaluate the differential expression level of the key genes, the Mann–Whitney *U* test was performed, and a two-tailed exact P-value of < 0.05 was considered statistically significant. Log-rank (Mantel-Cox) test was used for survival analysis, and a P-value of < 0.05 was considered to indicate a significant difference, the hazard ratio was calculated by the log-rank test. Two-way ANOVA with Sidak's multiple comparisons test was used to evaluate the therapeutic value of NVP-AUY922, P-values < 0.05 were considered statistically significant.

## Results

### Identification of DEGs and meta-analysis of three datasets

**Fig. 1:**
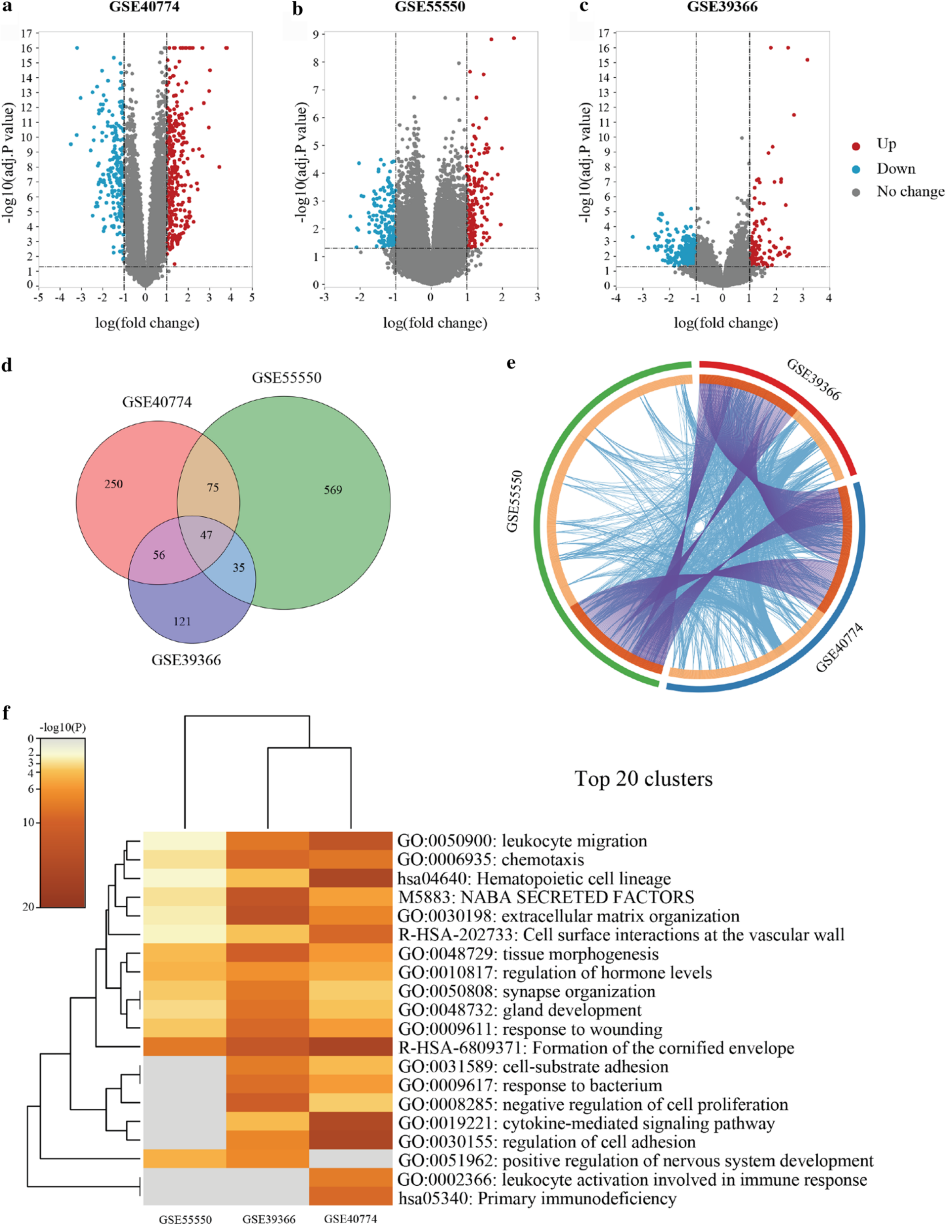
DEGs in three datasets from GEO and meta-analysis results. **a**–**c** Volcano plots showing the DEGs in GSE40774, GSE55550 and GSE39366, respectively. The red, blue and gray dots indicate genes with upregulated, downregulated, and unchanged expression, respectively, as determined by the thresholds. **d** Venn diagram showing the overlapping DEGs in the three datasets. **e** Meta-analysis showed the overlap between the gene lists: the purple curves connect identical genes, and the blue curves connect genes that belong to the same enriched GO term. The inner circle indicates the gene lists, and hits are arranged along the arc. Genes identified as hits in multiple lists are colored in dark orange, and genes unique to a list are colored in light orange. **f** Heatmap of the top 20 enriched GO terms across DEG lists from the meta-analysis of the three datasets. GO terms are colored by P-value

Three GEO datasets (GSE39366, GSE40774, GSE55550) containing a total of 345 samples were analyzed by GEO2R, with the data divided into the HPV-positive and HPV-negative groups. The details of the three datasets was summarized in Table 1. DEGs were identified as genes meeting the following criteria: adj.P-value < 0.05 and |log(FC)|≥ 1. The screen identified 259 DEGs in GSE39366, 428 in GSE40774 and 726 in GSE55550, and 47 overlapping genes were identified across the three datasets (Fig. 1a–d). Metascape was used to perform a meta-analysis on the DEGs from three datasets. The overlapping DEGs and the top 20 enriched GO terms were visualized in Circos plot and heatmap, respectively, and the most enriched GO term was leukocyte migration (Fig. 1e, f).

**Table 1:** Details of three GEO datasets

Reference (year)	Platform	Series	Samples	HPV (+)	HPV (−)
Walter V et al. (2013)	GPL9053	GSE39366	HNSCC	14	82
Keck MK et al. (2015)	GPL13497	GSE40774	HNSCC	58	76
Tomar S et al. (2016)	GPL17077	GSE55550	OPSCC	35 (24 HPV-inactive samples excluded)	80

*HNSCC* head and neck squamous cell carcinoma, *OPSCC* oropharyngeal squamous cell carcinoma

### GO and KEGG enrichment analysis of the 47 overlapping DEGs

**Fig. 2:**
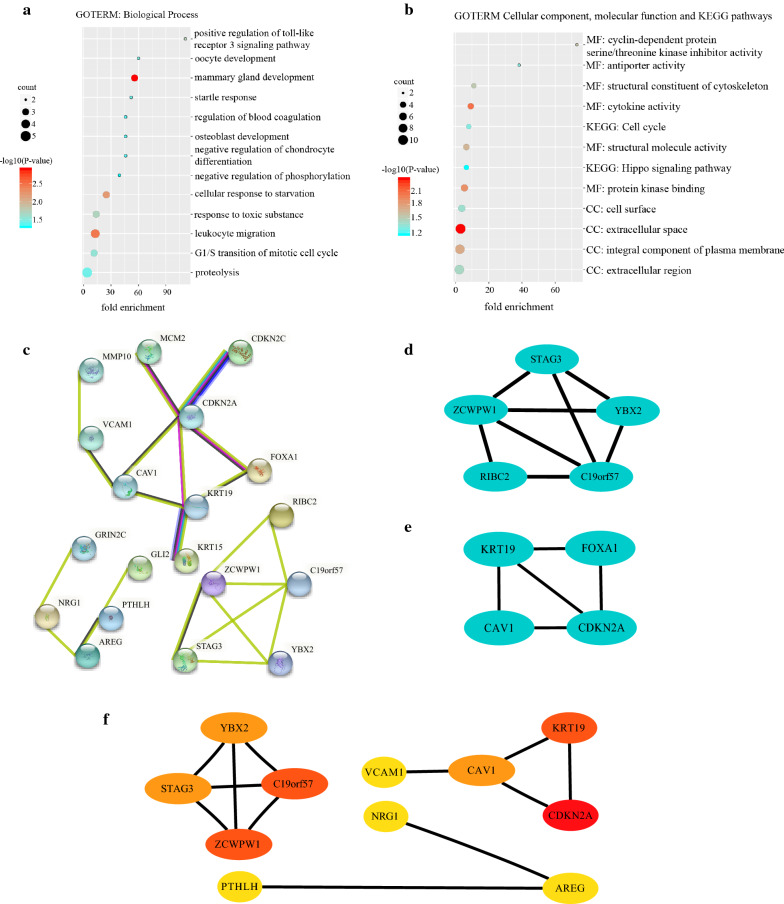
GO and KEGG enrichment analysis, PPI network construction, module analysis and hub gene identification. **a** Enriched biological process (BP) pathways. **b** Enriched cellular component (CC), molecular function (MF) and KEGG pathways. **c** PPI network of the 47 DEGs, constructed via the STRING database. **d**–**e** Top two most significant modules screened with the Cytoscape plugin MCODE. **f** Eleven hub genes identified with the Cytoscape plugin cytoHubba

DAVID was used to further analyze the BP and functional pathways enriched with the DEGs. The main enriched BPs were mammary gland development and leukocyte migration, similar to the results of the meta-analysis of all DEGs described above. In addition, positive regulation of the Toll-like receptor 3 signaling pathway was associated with pathogen recognition and activation of innate immunity, while the G1/S transition of the mitotic cell cycle was associated with carcinogenesis. These results showed that the DEGs are essential in tumor initiation and development (Fig. 2a). CC analysis indicated that the DEGs were most enriched in the extracellular space. Regarding the MF classification, the DEGs were enriched mainly in cytokine activity and protein kinase binding. KEGG analysis indicated that the DEGs were enriched mainly in cell cycle and Hippo signaling pathways, which regulate cell proliferation and apoptosis (Fig. 2b).

### Construction of the PPI network and identification of hub genes

**Fig. 3:**
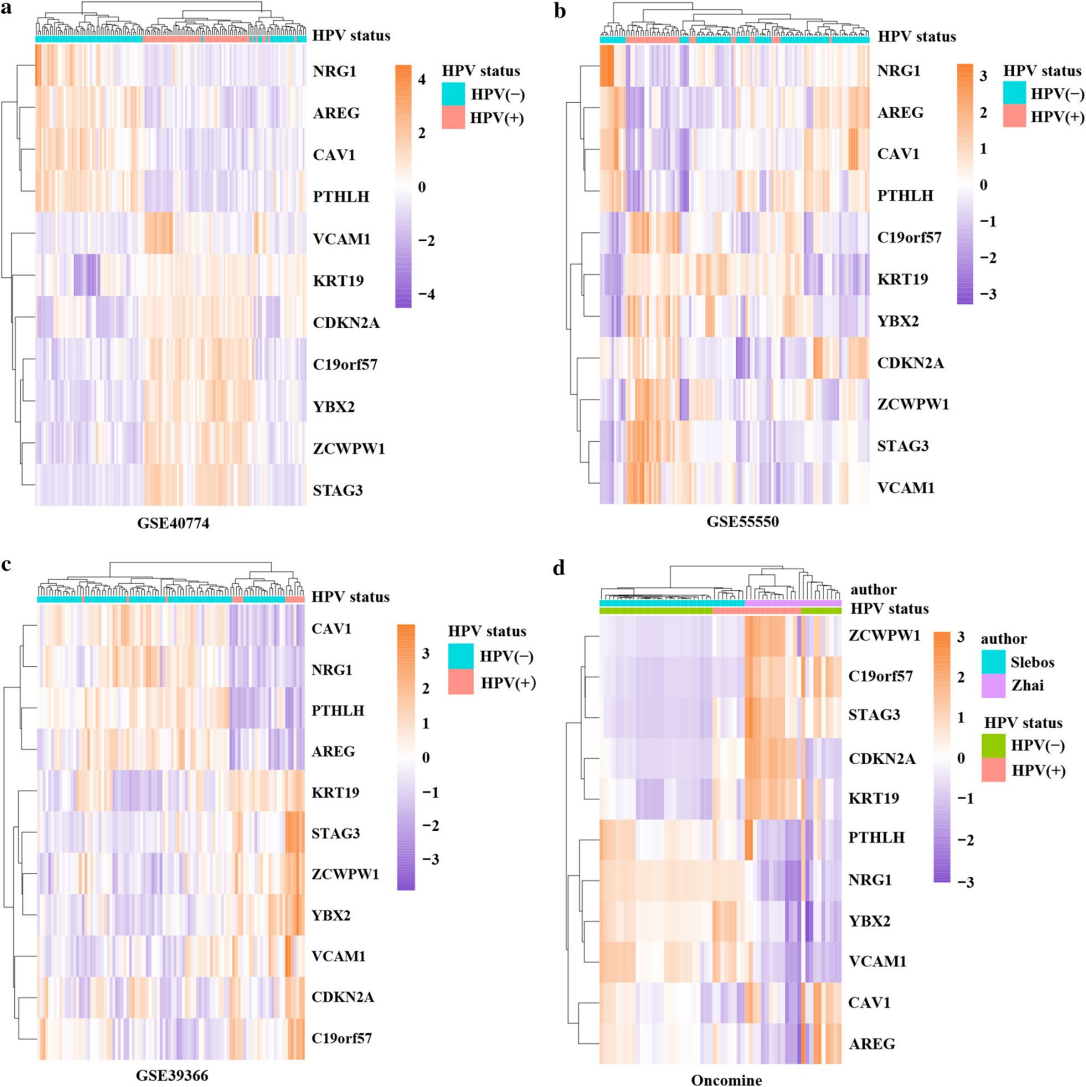
Heatmaps of hub genes from the three GEO datasets and verification via Oncomine database analysis. **a**–**c** Heatmaps of hub gene expression in the three GEO datasets. **d** Heatmap of genes in two Oncomine datasets used to verify the expression of the hub genes

STRING database was used to construct the PPI network between DEGs (Fig. 2c). The network included 47 nodes and 23 edges, and the PPI enrichment P-value was 4.91e-05. The top 2 significant modules were identified (Fig. 2d, e). Furthermore, the hub genes were screened with cytoHubba, and since 4 genes occupied the same rank, 11 genes were finally identified as hub genes (Fig. 2f). CDKN2A was the top-ranked gene, followed by C19orf57, ZCWPW1 and KRT19. The expression levels of these 11 hub genes in the GEO datasets were visualized with heatmaps (Fig. 3a–c). NRG1, AREG, CAV1 and PTHLH were downregulated in HPV-positive HNSCCs, while the remaining genes were upregulated. The opposite pattern was observed in HPV-negative HNSCC samples.

### Verification of hub gene expression patterns at the mRNA level based on Oncomine and TCGA data

**Fig. 4:**
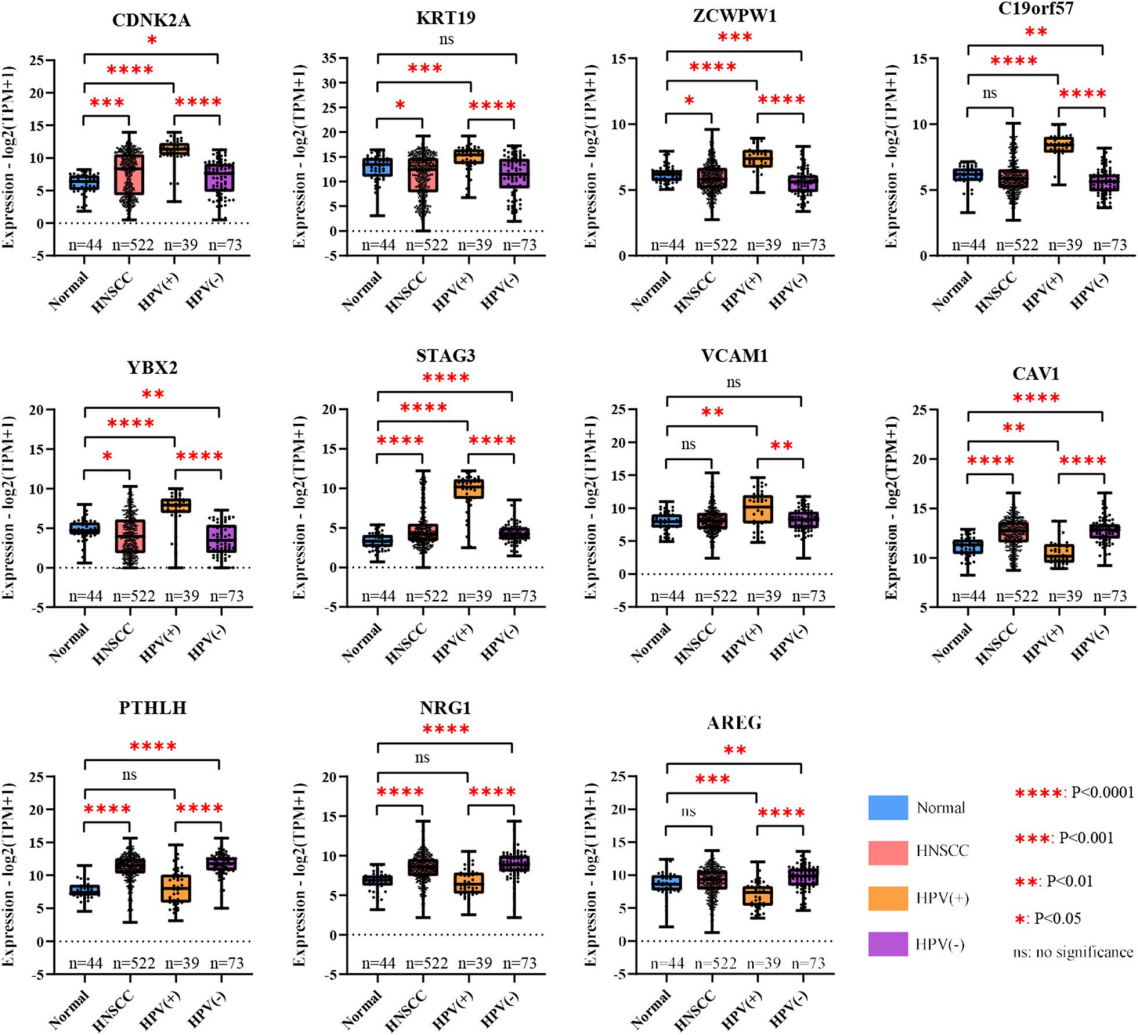
Boxplots showing mRNA expression of hub genes in normal tissues, HNSCCs, HPV-positive and HPV-negative HNSCCs based on TCGA datasets. The Mann–Whitney *U* test was performed, and a two-tailed exact P-value of < 0.05 was considered statistically significant

To verify the expression of the hub genes, two datasets from the Oncomine database were analyzed (Fig. 3d). The four genes identified as downregulated in the three GEO datasets also had lower expression levels in Oncomine datasets. In addition, TCGA data were downloaded to confirm the mRNA expression patterns of the key genes between HPV-positive and HPV-negative HNSCCs as well as between HNSCC and normal tissues. The results confirmed that all 11 hub genes were significantly differentially expressed between HPV-positive and -negative HNSCC tissues. In addition, similarly, CAV1, PTHLH, NRG1 and AREG were downregulated but the other genes were upregulated in HPV-positive HNSCCs (Fig. 4).

### The expression patterns of hub genes at the protein level

**Fig. 5:**
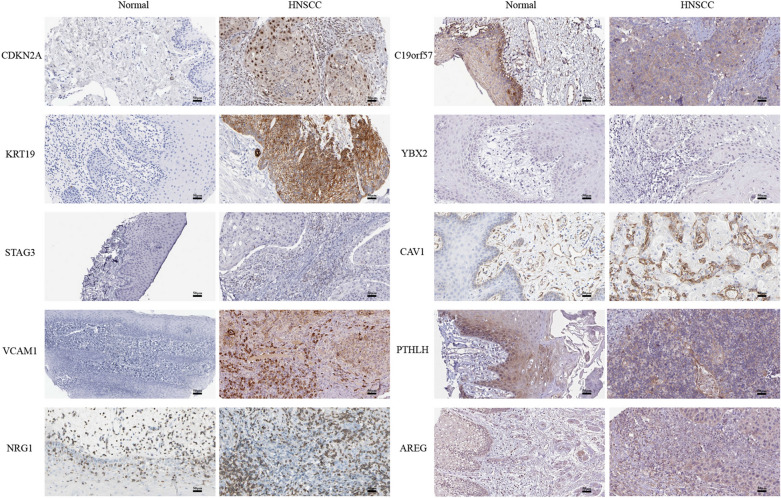
The expression of hub genes in HPA at the protein level, as shown through immunohistochemical analysis

The expression of hub genes at the protein level was investigated in the HPA. Since the HPV status was not available in the HPA, the expression patterns of ten key genes between HNSCCs and normal samples were analyzed by immunohistochemistry. Except for the result of ZCWPW1 staining, which was not provided, as shown in Fig. 5, the protein expression levels of CDKN2A, KRT19, VCAM1 and CAV1 in HNSCCs were higher than those in normal tissues, although staining for C19orf57 was enhanced in normal tissues. In addition, no differences were observed for STAG3, NRG1, PTHLH and AREG staining. These results indicated that the protein expression levels of the hub genes between HNSCCs and normal tissues were consistent with their mRNA expression levels.

### AREG, STAG3, C19orf57 and CAV1 were associated with the prognosis of HNSCC

**Fig. 6:**
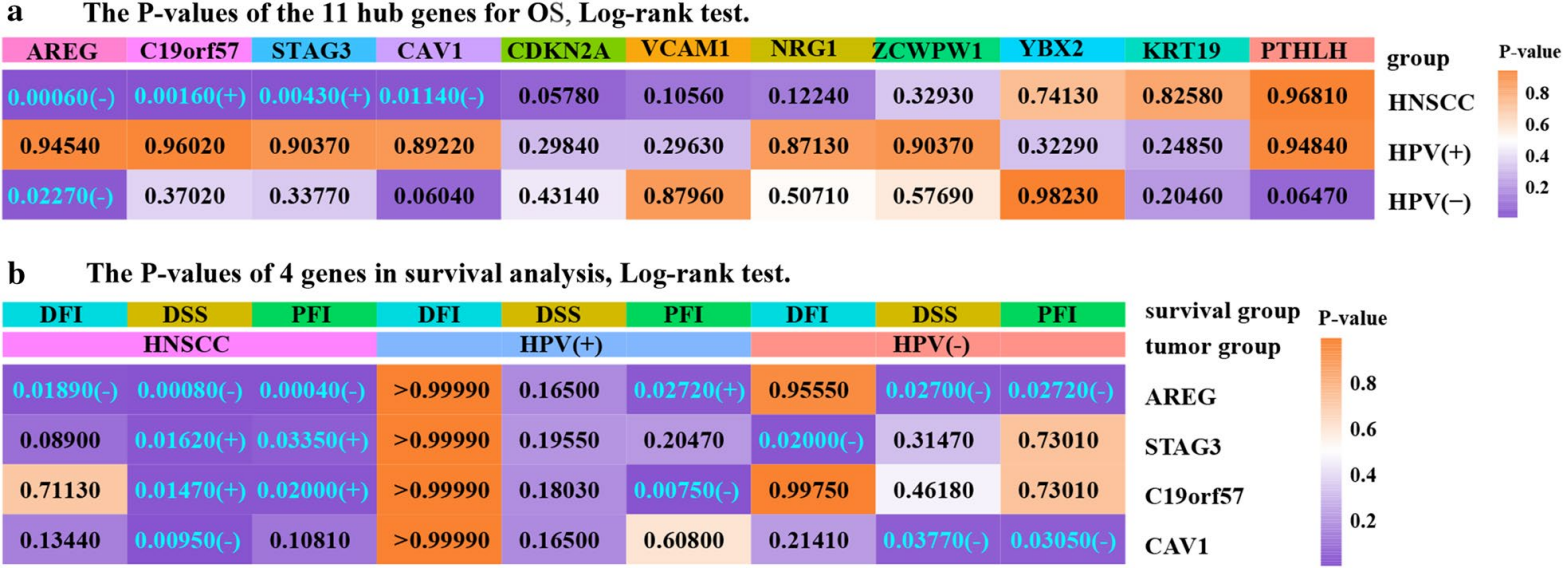
Over view of P-values in survival analysis. **a** Overview of the P-values of the 11 hub genes for OS. **b** Overview of the P-values of four genes in survival analysis. For the P-values, blue font indicates statistical significance. [“(+)” in blue means that the gene is a favorable prognostic factor, while “(−)” means that it is an unfavorable prognostic factor]

**Fig. 7:**
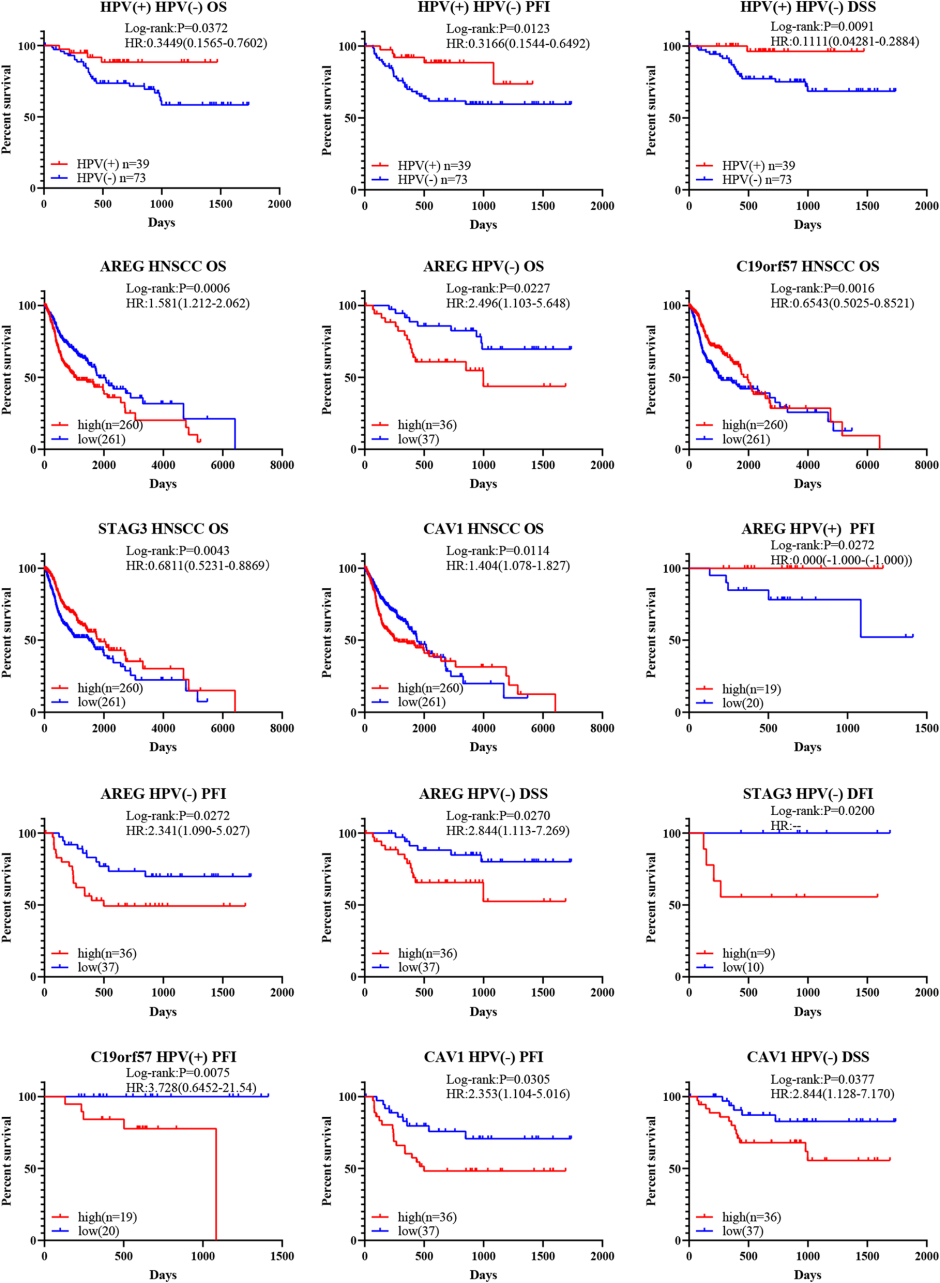
Survival analysis of patients stratified by HPV status and hub gene expression in HNSCC, HPV-positive and HPV-negative HNSCC tissues. The overall survival (OS), disease-free interval (DFI), disease-specific survival (DSS) and progression-free interval (PFI) of patients stratified by the expression levels of AREG, C19orf57, STAG3 and CAV1 were showed (Log-rank test, P < 0.05)

To further explore the prognostic value of the hub genes, survival analysis was performed. First, the OS, DFI, DSS and PFI of HPV-positive and HPV-negative patients were compared. HPV-positive patients exhibited higher OS rates, longer PFI times and higher DSS rates (Fig. 7; Additional file 1: Figure S1). Then, OS analysis of patients stratified by the expression levels of the 11 key genes revealed that AREG and CAV1 were associated with poor prognosis in HNSCC patients, C19orf57 and STAG3 were associated with favorable prognosis, and the other 7 genes were not significantly associated with OS (Additional file 2: Figure S2). Interestingly, AREG was also a prognostic factor in HPV-negative patients, and the OS rate was higher for patients with lower AREG expression levels (Figs. 6a, 7). Finally, DFI, DSS and PFI were further analyzed for patients stratified by the expression levels of the four genes identified as statistically significant in the OS analysis (Fig. 7; Additional file 3: Figure S3). These results indicated that the AREG gene had the highest prognostic value for DFI, DSS and PFI in patients with HNSCC and that it was a prognostic factor for favorable PFI in HPV-positive patients. Additionally, for HPV-negative patients, AREG could be a prognostic factor for unfavorable DSS and PFI. Moreover, STAG3 and C19orf57 were prognostic factors for favorable DSS and PFI in HNSCCs. STAG3 was the only prognostic factor for unfavorable DFI in HPV-negative patients, while C19orf57 could be used as an indicator of poor PFI in HPV-positive patients. Similar results were found for CAV1, which was a factor for unfavorable DSS in HNSCCs as well as for unfavorable DSS and PFI in HPV-negative patients (Figs. 6b, 7; Additional file 1: Figure S1). Overall, AREG, STAG3, C19orf57 and CAV1 were the four key factors with high prognostic value, and an overview of the P-values is shown in Fig. 6.

### AREG, CAV1 and STAG3 were associated with pathways dysregulated in cancer

**Fig. 8:**
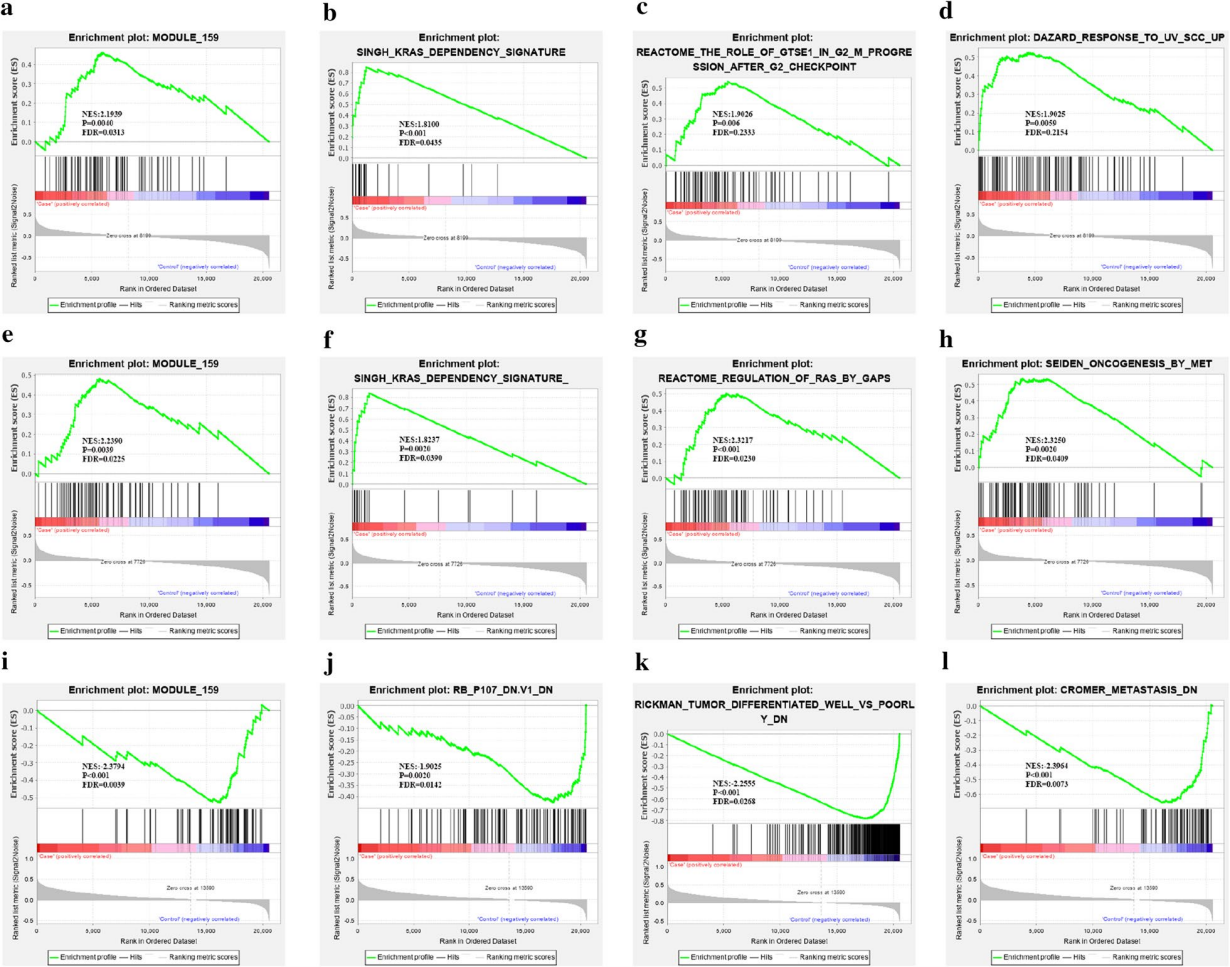
GSEA of computational gene sets, oncogenic gene sets and curated gene sets in TCGA samples with high expression vs low expression of AREG, CAV1 and STAG3. **a**–**d** The upregulation of AREG was associated with module 159 (translation regulation), the KRAS dependency signature, GTSE1 in G2/M progression after the G2 checkpoint, and genes upregulated in SCC12B2 cells (squamous cell carcinoma) by UV-B irradiation. **e**–**h** The upregulation of CAV1 was associated with module 159 (translation regulation), the KRAS dependency signature, regulation of RAS by GAPs, and oncogenesis by MET. **i**–**l** The downregulation of STAG3 was associated with module 159 (translation regulation), RB and P107 downregulation, poor differentiation and metastasis of HNSCC

AREG, CAV1 and STAG3 were associated with module 159 in our study (Fig. 8a, e, i). Module 159 is a translation regulation module in cancer and is defined by mining a large collection of tumor-oriented microarray data. AREG and CAV1 upregulation was related to the KRAS dependency signature in the collection of oncogenic signatures (Fig. 8b, f). In addition, AREG upregulated samples were enriched in the pathways of protein G-2 and S-phase expressed 1 (GTSE1) in G2/M progression after the G2 checkpoint (Fig. 8c), and associated with genes up-regulated in SCC12B2 cells (squamous cell carcinoma) by ultraviolet B (UV-B) irradiation (Fig. 8d). Increased CAV1 levels were associated with the regulation of the guanosine triphosphatase (GTPase) activity of RAS protein, which is stimulated by GTPase-activating proteins (GAPs) (Fig. 8g). In addition, CAV1 upregulation was related to oncogenesis regulated by Met (hepatocyte growth factor receptor) overexpression (Fig. 8h). STAG3 downregulation was linked to the retinoblastoma gene (RB) and P107 down regulation was associated with metastasis and poor differentiation of HNSCC (Fig. 8j–l). However, no enriched pathways met the cutoff criteria when C19orf57 was analyzed.

### Ten Small molecules maybe candidate drugs for HNSCC

**Fig. 9:**
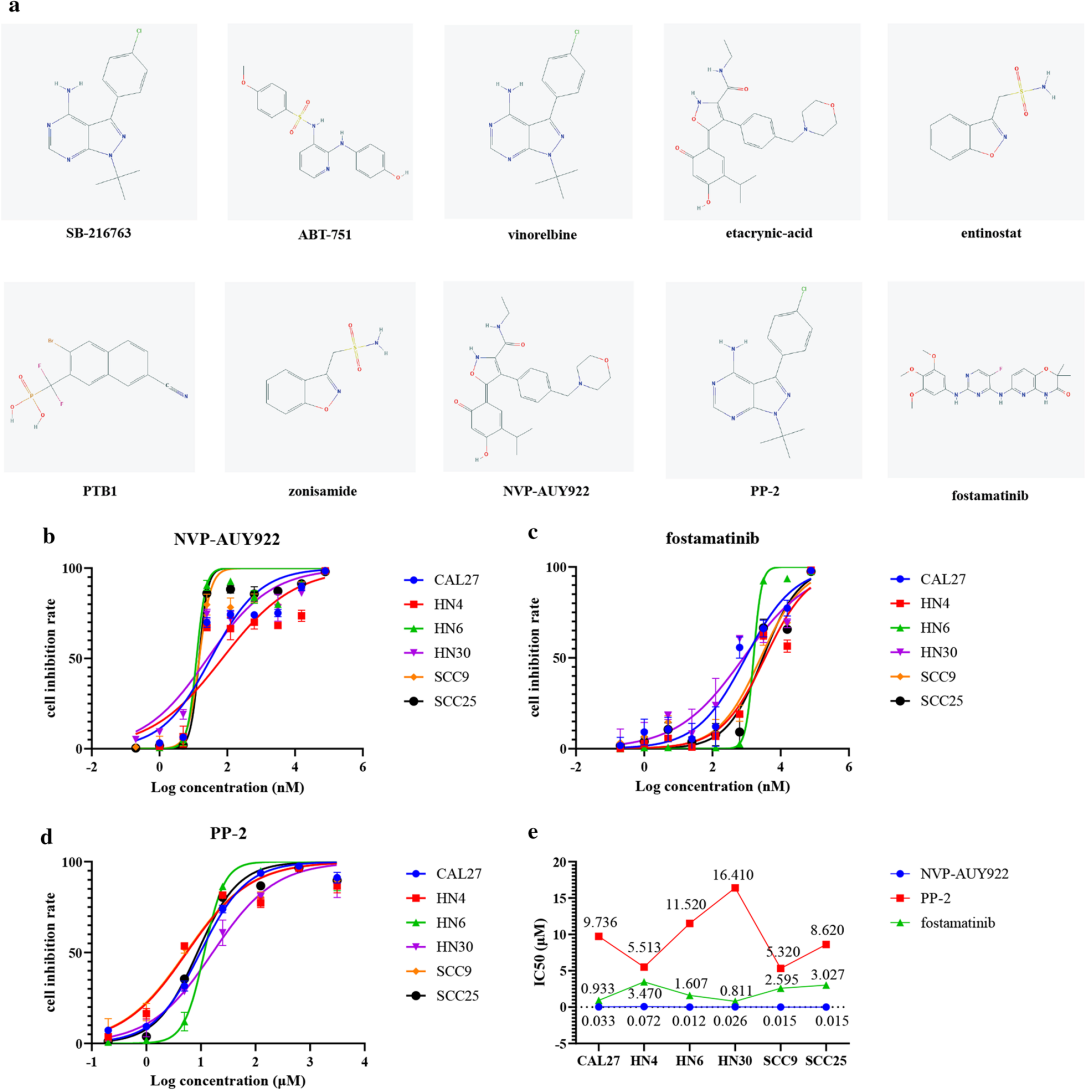
2D structure of the 10 small molecule drugs and dose–response curves (DRCs) of NVP-AUY922, fostamatinib and PP-2. **a** 2D structure of the 10 small molecule drugs. **b**–**d** DRCs of NVP-AUY922, fostamatinib and PP-2 in six cell lines, as determined by CCK8 assays. HN4, HN6, HN30, SCC9, SCC25 and CAL27 cell lines were seeded in 96-well culture plates, and the four drugs at different concentrations were added 24 h latter. After culture for 72 h, OD values were obtained by CCK8 assay, and cell inhibition rate was calculated. The experiments were repeated three times. **e** IC50 values of three small molecule drugs in six HPV-negative HNSCC cell lines. IC50 values of the drugs were calculated with GraphPad Prism software, NVP-AU922 was the most effective drug, followed by fostamatinib and PP-2

To further screen potential drugs for HNSCC, the up- and downregulated DEGs were uploaded to run the query. The molecules with the top five positive and negative scores were selected for further study. The details of these molecules were provided in Table 2. The molecules comprised 8 inhibitors, a blocker and an activator. A positive score indicated a similarity between a given perturbagen’s signature and that of the query, while a negative score indicated a disparity between the two signatures. In other words, molecules with a positive score may suppress HPV-negative HNSCCs, while molecules with a negative score may suppress HPV-positive HNSCCs. Further studies may confirm the value of these molecules. In addition, the 2D structures of the molecules are shown in Fig. 9a.

**Table 2:** Ten most significant small molecule drugs

Name	PubChem CID	Target	MOA	Score
SB-216763	176,158	GSK3B, CCNA2, CDK2, GSK3A	Glycogen synthase kinase inhibitor	− 98.08
ABT-751	3,035,714	TUBB	Tubulin inhibitor	− 97.94
Vinorelbine	73,707,424	TUBB	Tubulin inhibitor	− 97.78
Etacrynic-acid	3278	SLC12A1, ATP1A1, SLC12A2	Sodium/potassium/chloride transporter inhibitor	− 97.1
Entinostat	4261	HDAC1, HDAC2, HDAC3, HDAC9	HDAC inhibitor	− 95.33
PTB1	24,857,885	PTPN1	AMPK activator	99.08
Zonisamide	5734	CA1, CA12, CA7, SCN1A, CA10, CA11, CA13, CA14, CA2, CA3, CA4, CA5A, CA5B, CA6, CA8, CA9, CACNA1G, CACNA1H, CACNA1I, MAOA, MAOB, SCN11A, SCN1B, SCN2A, SCN2B, SCN3A, SCN3B, SCN4A, SCN4B, SCN5A, SCN9A	Sodium channel blocker, T-type calcium channel blocker	95.23
NVP-AUY922	10,096,043	HSP90AA1, HSP90AA2, HSP90AB1	HSP inhibitor	93.61
PP-2	4878	SRC, LCK, ABL1, LYN, RIPK2	SRC inhibitor	93.03
Fostamatinib	11,213,558	SYK, FLT3, RET	SYK inhibitor	92.89

### NVP-AUY922, fostamatinib, PP-2 inhibited cell proliferation in six HPV-negative HNSCC cell lines

Four candidate small molecule drugs (NVP-AUY922, fostamatinib, PP-2 and zonisamide) were evaluated the therapeutic value in vitro in six HPV-negative HNSCC cell lines. The proliferation of all cell lines was inhibited by each of the four drugs, and the proliferation inhibition rate was negatively associated with the drug concentration (Fig. 9b–d). NVP-AUY922 was the most effective drug, followed by fostamatinib and PP-2. The IC50 values of NVP-AUY922, fostamatinib and PP-2 were range from 0.012 to 0.072 μM, 0.811 to 3.470 μM, 5.32 to 16.41 μM, respectively (Fig. 9e). However, zonisamide may not be therapeutically effective, because its IC50 ranged from 86.46 to 150.5 μM (Additional file 4: Figure S4).

### NVP-AUY922 inhibited HPV-negative HNSCC xenografts

**Fig. 10:**
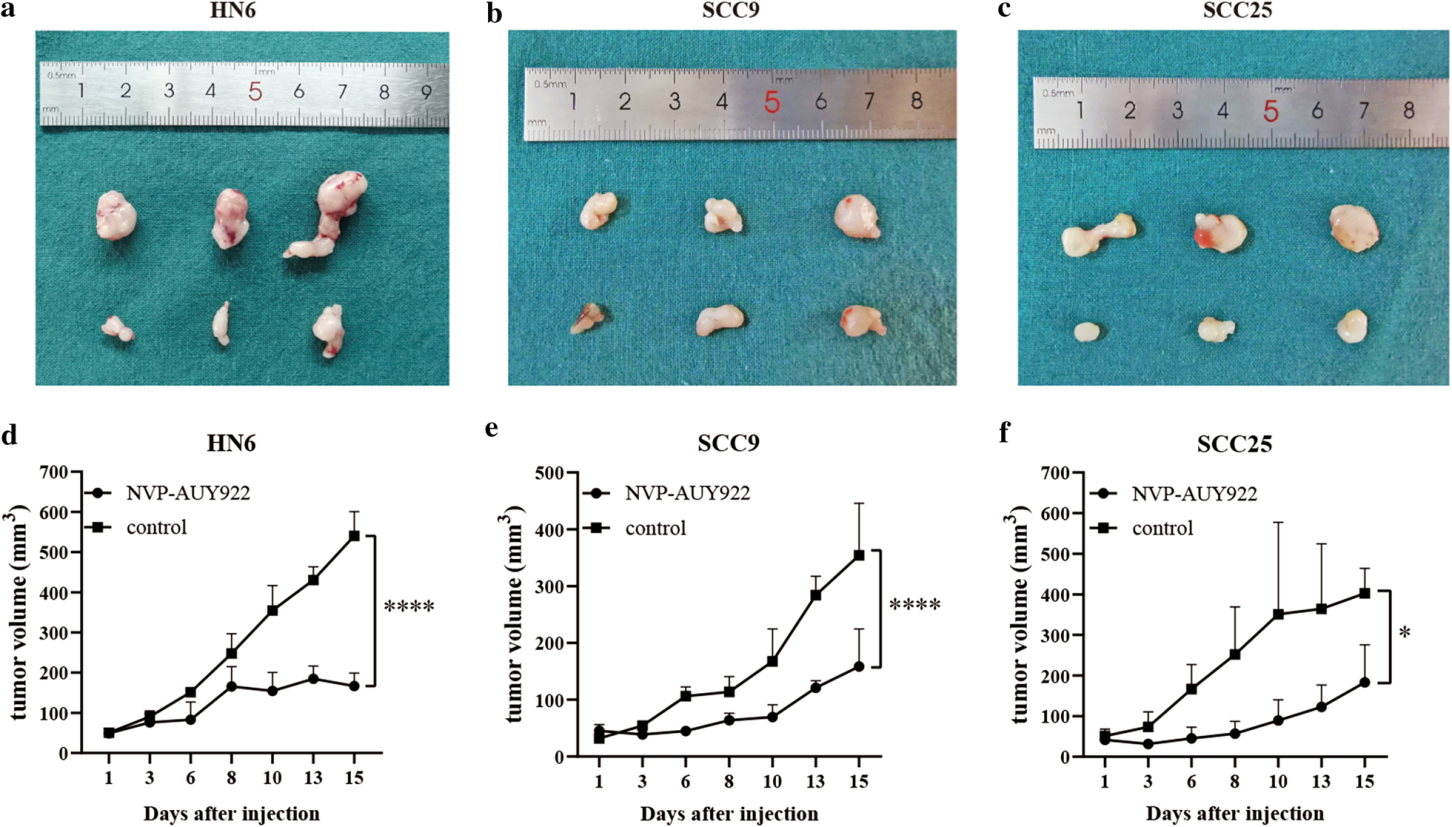
NVP-AUY922 inhibited three HPV-negative HNSCC xenografts in vivo. **a**–**c** The pictures showed tumor size of NVP-AUY922 treated group and control group in HN6, SCC9 and SCC25 xenografts. **d**–**f** The tumor growth curve of NVP-AUY922 treated and control group in three HPV-negative HNSCC xenografts. ****P < 0.0001, ***P < 0.001, **P < 0.01, *P < 0.05 (two-way ANOVA, Sidak’s multiple comparisons test)

To test the therapeutic effect in vivo, the most effective drug (NVP-AUY922) and three cell lines (HN6, SCC9, SCC25) were selected for further research according to the results in vitro (Fig. 9e). The HNSCC xenografts were established in BALB/c nude mice, and treated with 50 mg/kg NVP-AUY922 three times a week for two weeks. As a result, NVP-AUY922 significantly inhibited the growth of all the three HNSCC xenografts in vivo compared with the control group (Fig. 10a–f).

## Discussion

In this study, AREG, STAG3, C19orf57 and CAV1 were identified and showed different prognostic values for OS, DFI, DSS and PFI in HPV-positive and -negative patients. And GSEA revealed AREG, CAV1 and STAG3 were associated with dysregulated pathways in cancer. In addition, ten small molecules were screened as potential drugs for HNSCC. Three of them, NVP-AUY922, PP-2 and fostamatinib showed therapeutic value in six HPV-negative HNSCC cell lines, besides, NVP-AUY922 inhibited three HPV-negative HNSCC xenografts in vivo.

AREG is a ligand of the epidermal growth factor receptor (EGFR), AREG binds to EGFR can induce key intracellular signaling cascades controlling cell survival, proliferation and motility [27]. AREG is associated with several tumors, such as lung, breast, colorectal, ovary and prostate carcinomas, due to its role in tumorigenesis [28]. In a clinical trial of patients with HNSCC, patients with higher expression of AREG had shorter OS and PFI than patients with lower expression of AREG [29]. In addition, a recent study indicated vincristine resistance is promoted by AREG in oral squamous carcinoma [30]. Notably, in our study, AREG was a prognostic factor for unfavorable PFI and DSS in HPV-negative patients, but a prognostic factor for favorable PFI in HPV-positive patients. These results indicated that AREG may be strongly associated with the HPV status and is a potential therapeutic target in HNSCC.

STAG3 is a subunit of the cohesin complex that regulates the cohesion of sister chromatids during cell division. STAG3 is also required for chromosome pairing and synapsis, DNA repair and meiosis progression, and mutation of STAG3 may induce DNA repair process abnormalities [31]. A previous study demonstrated that STAG3 may be a tumor suppressor gene, and that loss of STAG3 may cause drug resistance in melanoma [32]. Here, a higher mRNA level of STAG3 was observed in HPV-positive HNSCC samples than in HPV-negative HNSCC samples, and STAG3 was suggested to be a biomarker for poor prognosis in HPV-negative patients. These outcomes were consistent with those of another study [33]. However, because of the small sample size in this study, more studies are needed to clarify the role of STAG3 in HPV-negative HNSCC patients.

Recently, C19orf57 was found to be significantly upregulated in HPV-active oropharyngeal squamous cell carcinoma (OPSCC) patients [34], indicating that C19orf57 may be a key gene associated with HPV. Similarly, our data support C19orf57 is a biomarker for unfavorable PFI times in patients with HPV-positive HNSCC. In addition, CAV1 is a major structural protein in caveolae and reported as an integral membrane protein associated with the progression of carcinoma. However, whether CAV1 act as an oncogene or a tumor suppressor gene in cancer progression is still unclear [35]. Several studies have indicated CAV1 is overexpressed in HNSCC and mediates tumor migration and invasion [36–38]. Similarly, we observed that CAV1 mRNA and protein levels were higher in HNSCC than in normal tissues, suggesting that CAV1 is a factor indicating poor OS. Thus, our results support the function of CAV1 as an oncogene.

Treatment of HNSCC remains challenging because approximately 67% of HNSCC patients present with advanced disease [4], and no effective drugs or suitable surgical methods are available. Thus, ten small molecules were screened as potential drugs in this study. SB-216763, ABT-751, vinorelbine, etacrynic acid and entinostat were suggested as candidate drugs for HPV-positive tumors, while PTB1, zonisamide, NVP-AUY922, PP-2 and fostamatinib were suggested as candidate drugs for HPV-negative tumors. NVP-AUY922, PP-2, and fostamatinib inhibited cell proliferation in vitro in six HPV-negative cell lines, with low IC50 values.

Heat shock proteins (HSPs) are chaperone proteins that can assist the folding of proteins associated with tumor growth. NVP-AUY922 is an HSP inhibitor that suppresses the growth, angiogenesis and metastasis of several kinds of tumors, including oral squamous cell carcinoma [39, 40]. These results support our finding that NVP-AUY922 was the most effective among the investigated drugs. Spleen tyrosine kinase (SYK) controls B-cell receptor (BCR) signal initiation and amplification, which promotes B-cell lymphoma progression [41]. A previous study reported fostamatinib, a SYK inhibitor, suppresses chronic lymphocytic leukemia [42] and prevents metastatic recurrence in breast cancer in vivo [43]. Fostamatinib has been approved for the treatment of immune thrombocytopenia autoimmune hemolytic anemia and IgA nephropathy [44], and the present study indicated that it may be useful in HPV-negative HNSCC patients. SRC is a non-receptor tyrosine kinase family member with a crucial role in tumor progression. PP-2, an inhibitor of SRC, may significantly ameliorate the invasiveness of breast cancer cells and enhance the radiosensitivity of glioma cells [45, 46]. Moreover, in our study, PP-2 showed therapeutic value in six HPV-negative HNSCC cell lines in vitro.

However, there may be some limitations in our study, such as the therapeutic value of the most significant small molecule drug PTB1 was not tested because it’s not available commercially, and therefore not able to obtain. Besides, NVP-AUY922 showed the most effective therapeutic value in vitro in six HPV-negative cell lines, so we further evaluated the therapeutic value of NVP-AUY922 in vivo, but more studies are needed to test if the other two significant drugs (PP-2 and fostamatinib) have better anti-tumor effect in vivo. Also, further studies both in vitro and in vivo should be performed to test the therapeutic value of the five significant drugs (Table 2) for HPV-positive HNSCC. What’s more, AREG, STAG3, C19orf57 and CAV1 are key prognostic biomarkers for HPV-negative HNSCC, they may also be potential therapeutic targets, drugs targeting the four key genes either alone or in combination may be new strategies for the treatment of HNSCC in further investigations.

In summary, our study identified four key genes (AREG, STAG3, C19orf57 and CAV1) and ten small molecules for the treatment of HNSCC. Three of the identified small molecule drugs (NVP-AUY922, PP-2 and fostamatinib) showed inhibitory effects on six HPV-negative HNSCC cell lines, and NVP-AUY922 inhibited three HPV-negative HNSCC xenografts in vivo. These genes may be therapeutic targets, and the small molecule drugs may be used alone or in combination with other drugs in further research aiming to improve the prognosis of patients.

## Conclusions

In conclusion, our study analyzed DEGs between HPV-positive and -negative HNSCCs. A total of 47 DEGs were screened, and 11 hub genes were identified as hub genes, among which AREG, STAG3, C19orf57 and CAV1 may be considered key prognostic biomarkers and therapeutic targets for HNSCC. In addition, NVP-AUY922, fostamatinib and PP-2 may be used as drugs to treat HPV-negative head and neck cancers.

## Supplementary Information

**Additional file 1: Figure S1.** Survival analysis of patients stratified by the expression of hub genes in HNSCC tissues.

**Additional file 2: Figure S2.** Overall survival analysis of patients stratified by the expression of hub genes in HNSCC, HPV-positive and HPV-negative HNSCC tissues.

**Additional file 3: Figure S3.** Survival analysis of the patients stratified by the expression of 4 genes in HNSCC, HPV-positive and HPV-negative HNSCC tissues.

**Additional file 4: Figure S4.** Dose response curve (DRC) and IC50 of zonisamide for six cell lines by CCK8 assay.

## Data Availability

The datasets generated and/or analyzed during the current study are available in GEO (http://www.ncbi.nlm.nih.gov/geo); University of California Santa Cruz Xena (https://xenabrowser.net/); Oncomine (https://www.oncomine.org/); The Human Protein Atlas (http://www.proteinatlas.org) and Connectivity Map (https://clue.io/cmap).

## Abbreviations

BCR: B-cell receptor
BP: Biological process
CC: Cellular component
CCK8: Cell counting kit-8
DAVID: Database for annotation, visualization and integrated discovery
DEG: Differentially expressed genes
DFI: Disease-free interval
DMSO: Dimethyl sulfoxide
DSS: Disease-specific survival
EGFR: Epidermal growth factor receptor
FDR: False discovery rate
GAPs: Guanosine triphosphatase activating proteins
GEO: Gene expression omnibus
GO: Gene ontology
GSEA: Gene set enrichment analysis
GTPase: Guanosine triphosphatase
GTSE1: G-2 and S-phase expressed 1
HNSCC: Head and neck squamous cell carcinoma
HPA: Human protein atlas
HPV: Human papillomavirus
HR: Hazard ratio
HSPs: Heat shock proteins
IC50: Half-maximal inhibitory concentrations
KEGG: Kyoto Encyclopedia of Genes and Genomes
MCODE: Molecular complex detection
MF: Molecular function
MSigDB: Molecular signatures database
OD: Optical density
OPSCC: Oropharyngeal squamous cell carcinoma
OS: Overall survival
PFI: Progression-free interval
PPI: Protein–protein interaction
STRING: Search tool for the retrieval of interacting genes/proteins
SYK: Spleen tyrosine kinase
TCGA: The cancer genome atlas
UCSC: University of California Santa Cruz
UV-B: Ultraviolet B
