# Correction to: Identification of four key prognostic genes and three potential drugs in human papillomavirus negative head and neck squamous cell carcinoma

**DOI:** 10.1186/s12935-021-01904-0

**Published:** 2021-05-04

**Authors:** Guocai Tian, You Fu, Dahe Zhang, Jiang Li, Zhiyuan Zhang, Xi Yang

**Affiliations:** 1 Department of Oral and Maxillofacial‑Head Neck Oncology, Shanghai Ninth, People’s Hospital, College of Stomatology, School of Medicine, Shanghai Jiao Tong University, Shanghai, People’s Republic of China; 2 National Clinical Research Center for Oral Diseases, Shanghai, People’s Republic of China; 3 Shanghai Key Laboratory of Stomatology and Shanghai Research Institute of Stomatology, Shanghai, People’s Republic of China; 4 Research Unit of Oral and Maxillofacial Regenerative Medicine, Chinese Academy of Medical Sciences, Shanghai, People’s Republic of China; 5 Department of Oral Pathology, Ninth People’s Hospital, Shanghai Jiao Tong University School of Medicine, Shanghai, People’s Republic of China

## Correction to: Cancer Cell Int (2021) 21:167 https://doi.org/10.1186/s12935-021-01863-6

Following the publication of the original article [1], the authors reported that they had supplied an incorrect Fig. 5 (the IHC staining of PTHLH in HNSCC sample), the original figure was from Human Protein Atlas (https://www.proteinatlas.org/ENSG00000087494-PTHLH/pathology/head+and+neck+cancer#img).

**Fig. 5:**
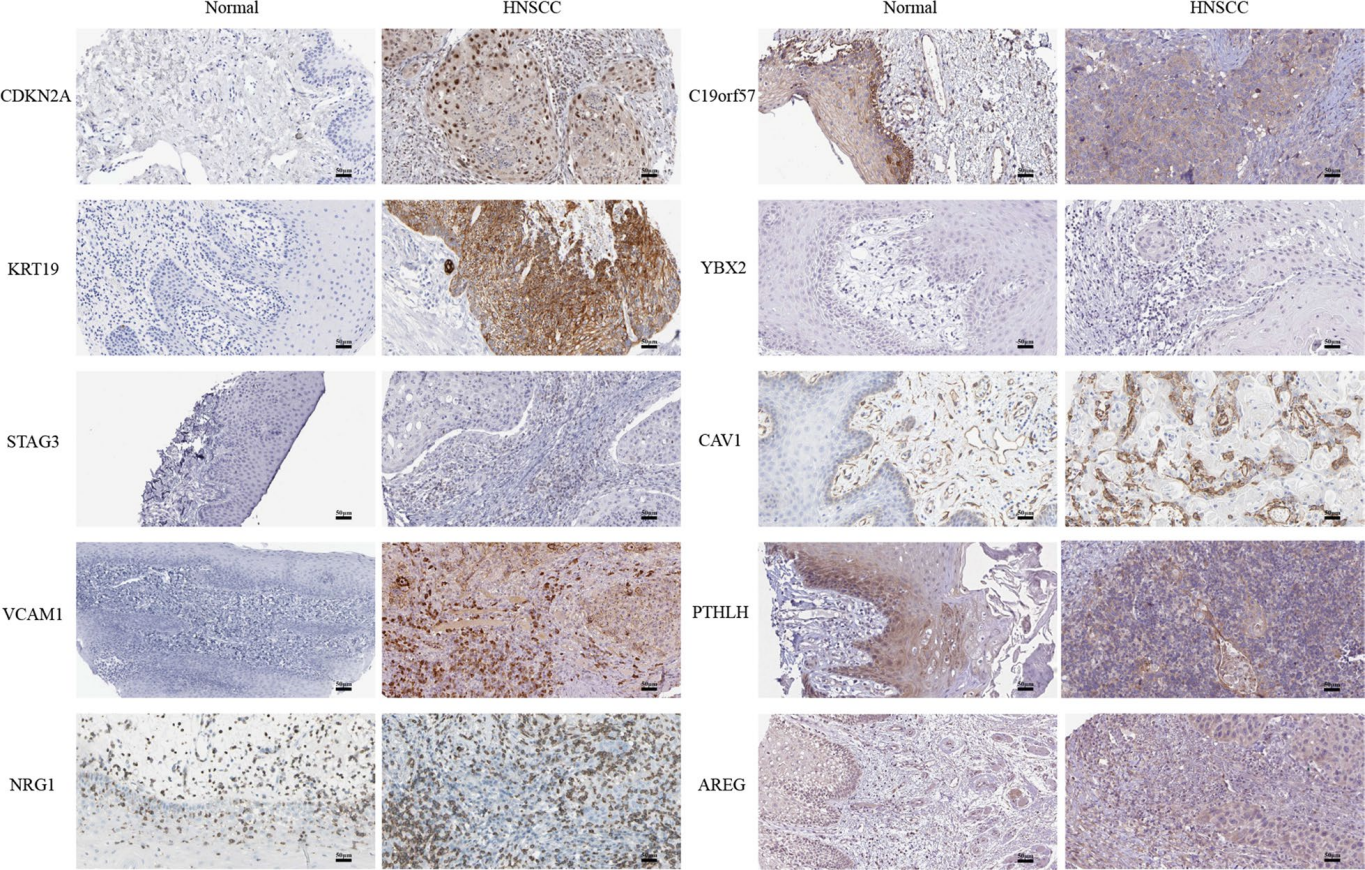
The expression of hub genes in HPA at the protein level, as shown through immunohistochemical analysis

The correct Fig. 5 is given above in this correction article. The results and conclusions described are not affected by these corrections. The authors sincerely apologize for the error.

The original article has been corrected.
