# Supplementary material for: Identification of four key prognostic genes and three potential drugs in human papillomavirus negative head and neck squamous cell carcinoma
Source: Cancer Cell Int. 2021 Mar 12;21:167. doi: 10.1186/s12935-021-01863-6 (PMC7953640; doi:10.1186/s12935-021-01863-6)
Supplement: Supplementary file 1 — Additional file 1: Figure S1. Survival analysis of patients stratified by the expression of hub genes in HNSCC tissues. [file 12935_2021_1863_MOESM1_ESM.pdf]

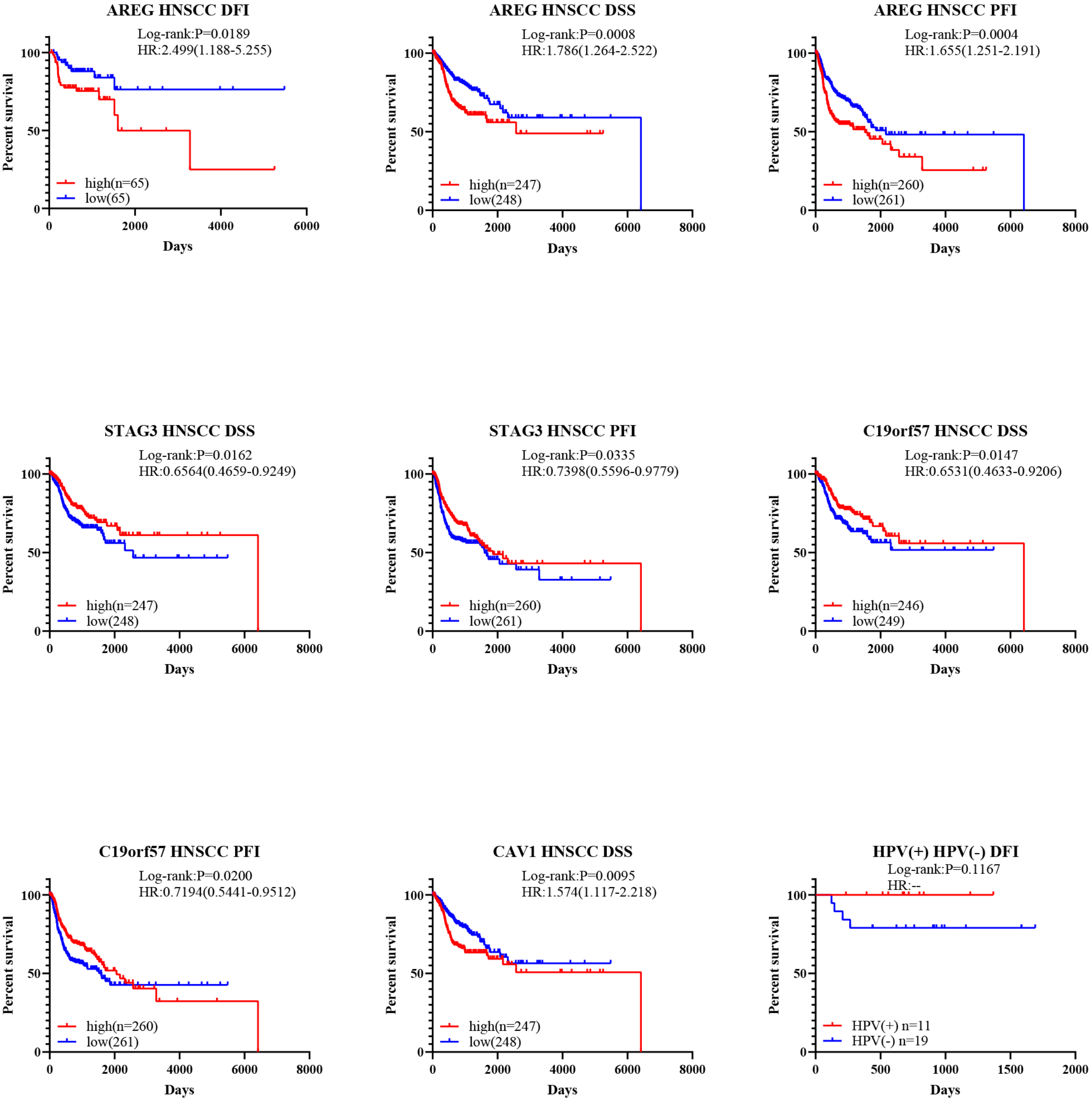
AREG HNSCC DFI
Log-rank:P=0.0189
HR:2.499(1.188-5.255)
high(n=65)
low(65)
AREG HNSCC DSS
Log-rank:P=0.0008
HR:1.786(1.264-2.522)
high(n=247)
low(248)
AREG HNSCC PFI
Log-rank:P=0.0004
HR:1.655(1.251-2.191)
high(n=260)
low(261)
STAG3 HNSCC DSS
Log-rank:P=0.0162
HR:0.6564(0.4659-0.9249)
high(n=247)
low(248)
STAG3 HNSCC PFI
Log-rank:P=0.0335
HR:0.7398(0.5596-0.9779)
high(n=260)
low(261)
C19orf57 HNSCC DSS
Log-rank:P=0.0147
HR:0.6531(0.4633-0.9206)
high(n=246)
low(249)
C19orf57 HNSCC PFI
Log-rank:P=0.0200
HR:0.7194(0.5441-0.9512)
high(n=260)
low(261)
CAV1 HNSCC DSS
Log-rank:P=0.0095
HR:1.574(1.117-2.218)
high(n=247)
low(248)
HPV(+) HPV(-) DFI
Log-rank:P=0.1167
HR:--
HPV(+) n=11
HPV(-) n=19
Percent survival
Days
