# Supplementary figures and images for: Identification of four key prognostic genes and three potential drugs in human papillomavirus negative head and neck squamous cell carcinoma
Source: Cancer Cell Int. 2021 Mar 12;21:167. doi: 10.1186/s12935-021-01863-6 (PMC7953640; doi:10.1186/s12935-021-01863-6)

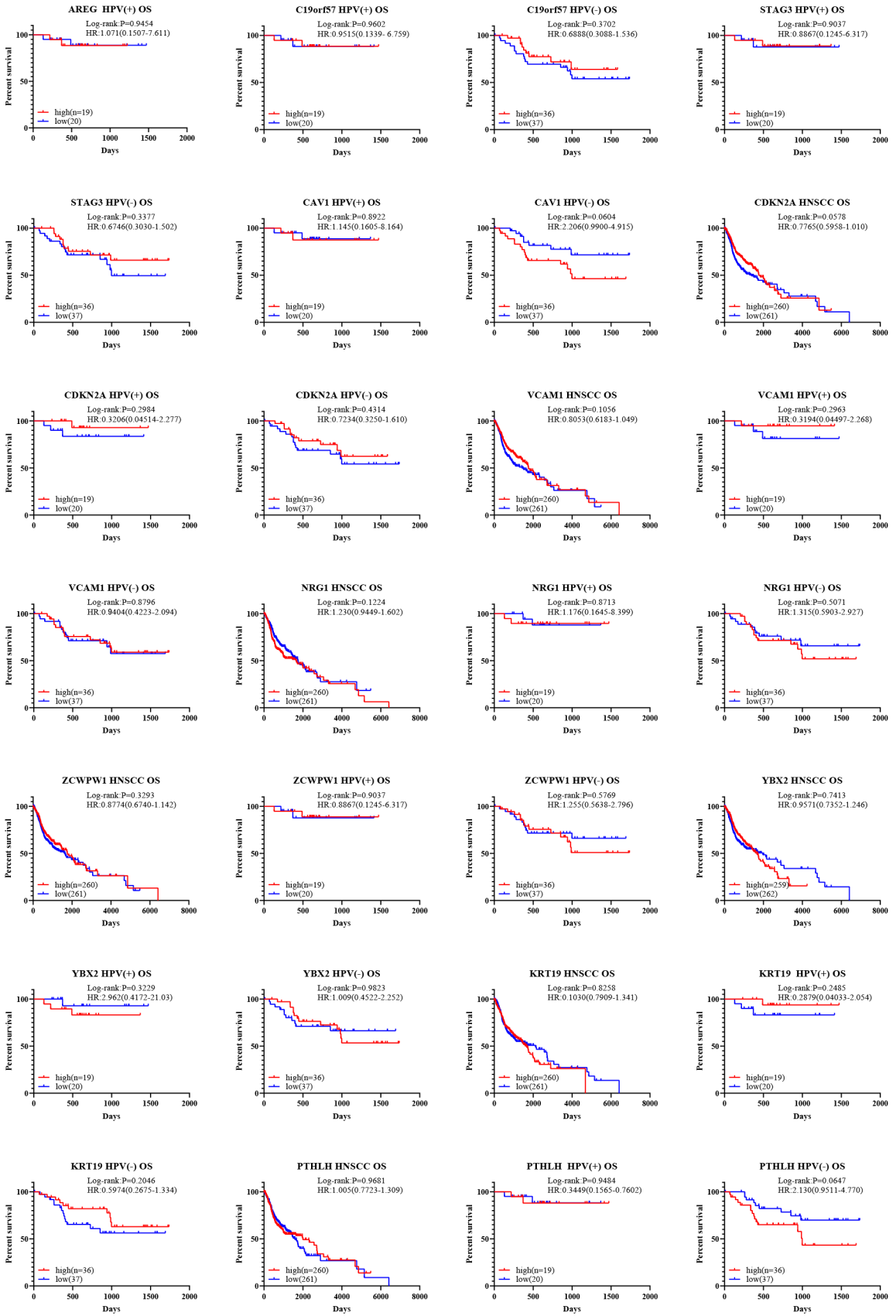

Supplement: Supplementary file 2 — Additional file 2: Figure S2. Overall survival analysis of patients stratified by the expression of hub genes in HNSCC, HPV-positive and HPV-negative HNSCC tissues. [file 12935_2021_1863_MOESM2_ESM.pdf]

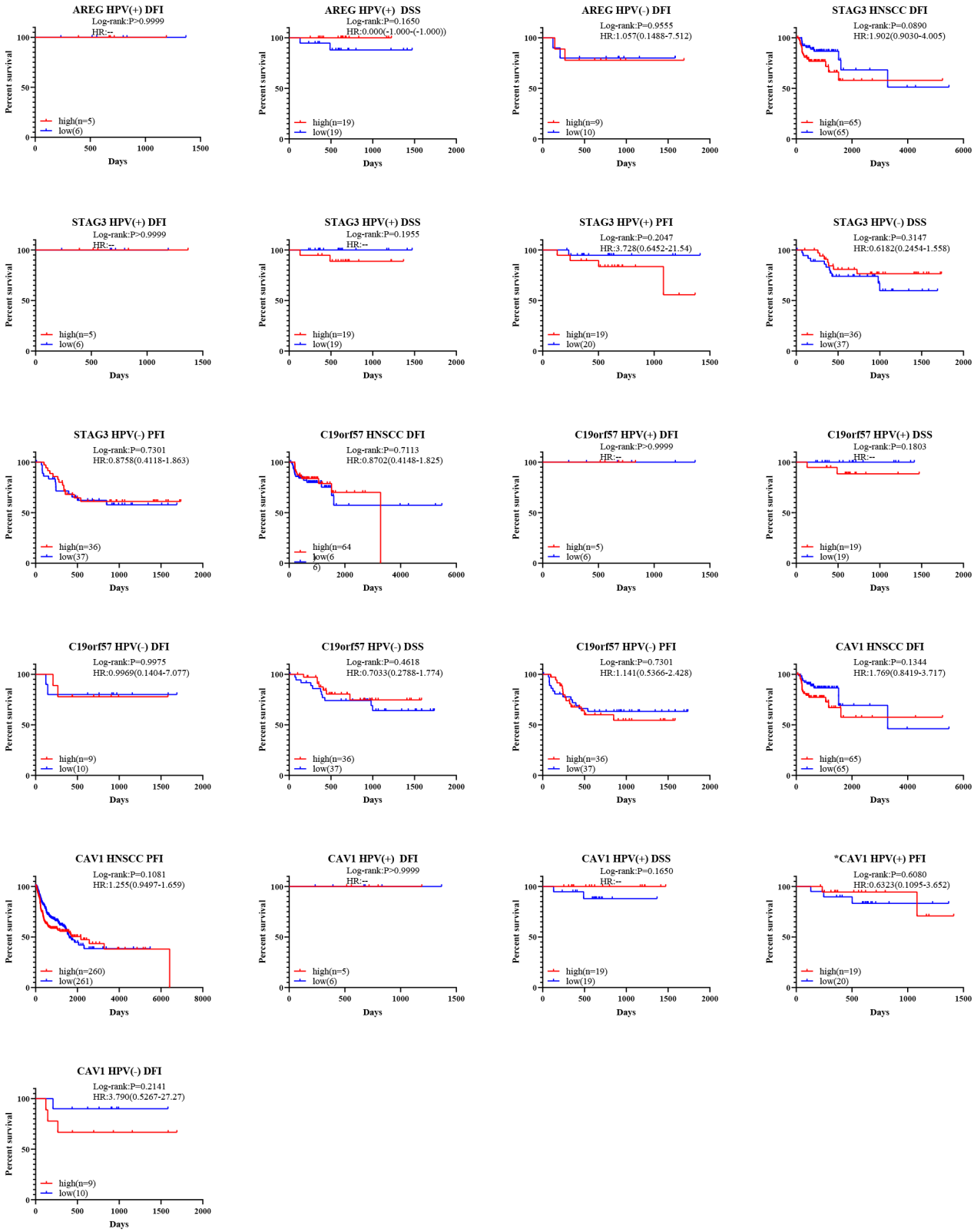

Supplement: Supplementary file 3 — Additional file 3: Figure S3. Survival analysis of the patients stratified by the expression of 4 genes in HNSCC, HPV-positive and HPV-negative HNSCC tissues. [file 12935_2021_1863_MOESM3_ESM.pdf]

**a****zonisamide**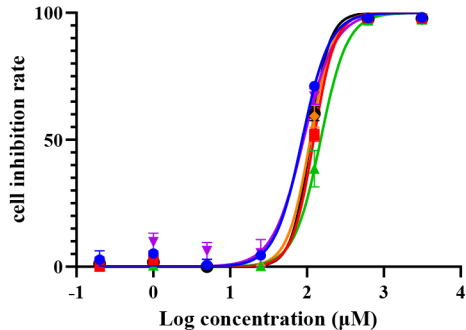**b**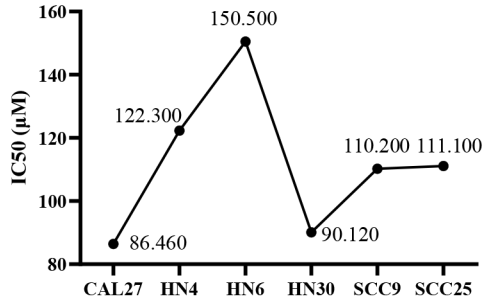

Supplement: Supplementary file 4 — Additional file 4: Figure S4. Dose response curve (DRC) and IC50 of zonisamide for six cell lines by CCK8 assay. [file 12935_2021_1863_MOESM4_ESM.pdf]
